# Bovine antibodies targeting primary and recurrent *Clostridium difficile* disease are a potent antibiotic alternative

**DOI:** 10.1038/s41598-017-03982-5

**Published:** 2017-06-16

**Authors:** Melanie L. Hutton, Bliss A. Cunningham, Kate E. Mackin, Shelley A. Lyon, Meagan L. James, Julian I. Rood, Dena Lyras

**Affiliations:** Infection and Immunity Program, Monash Biomedicine Discovery Institute and Department of Microbiology, Monash University, Clayton, Victoria 3800 Australia

## Abstract

The increased incidence of antibiotic resistant ‘superbugs’ has amplified the use of broad spectrum antibiotics worldwide. An unintended consequence of antimicrobial treatment is disruption of the gastrointestinal microbiota, resulting in susceptibility to opportunistic pathogens, such as *Clostridium difficile*. Paradoxically, treatment of *C. difficile* infections (CDI) also involves antibiotic use, leaving patients susceptible to re-infection. This serious health threat has led to an urgent call for the development of new therapeutics to reduce or replace the use of antibiotics to treat bacterial infections. To address this need, we have developed colostrum-derived antibodies for the prevention and treatment of CDI. Pregnant cows were immunised to generate hyperimmune bovine colostrum (HBC) containing antibodies that target essential *C. difficile* virulence components, specifically, spores, vegetative cells and toxin B (TcdB). Mouse infection and relapse models were used to compare the capacity of HBC to prevent or treat primary CDI as well as prevent recurrence. Administration of TcdB-specific colostrum alone, or in combination with spore or vegetative cell-targeted colostrum, prevents and treats *C*. *difficile* disease in mice and reduces disease recurrence by 67%. *C. difficile*-specific colostrum should be re-considered as an immunotherapeutic for the prevention or treatment of primary or recurrent CDI.

## Introduction

There is a desperate worldwide need to minimise the use of antibiotics. Although the treatment of most bacterial infections relies on antibiotic use, resistance has emerged and is now one of our most serious global health threats. Also of concern is the rapid increase in nosocomial antibiotic-associated infections caused by opportunistic pathogens such as *Clostridium difficile. C. difficile* infection (CDI) is most often associated with antibiotic use as the alteration to the endogenous gastrointestinal microbiota results in increased susceptibility to CDI^1^. The over-use of antibiotics has been a driver for the astonishing increase in the rate and prevalence of *C. difficile*, as has the change in the virulence of the causative strains, with BI/NAP1/027 isolates causing increased mortality rates in the UK, USA, Canada and Europe in the last decade^1^.

*C. difficile* is a Gram-positive, spore-forming, anaerobic bacterium that infects the gastrointestinal tract and causes an array of clinical symptoms ranging from mild diarrhoea to more severe, often fatal, gastrointestinal disease such as pseudomembranous colitis and toxic megacolon^2^. The infection cycle of *C*. *difficile* is complex because this bacterium produces spores that are highly resistant to environmental assaults, enabling persistence in unfavourable environments^3^. Spores are the infectious particles ingested by the host, where they germinate into vegetative cells, colonise the large intestine and establish infection^1^. Disease symptoms occur in response to toxin-mediated damage with up to three secreted toxins, TcdA, TcdB and CDT, variably produced by strains^1^. TcdA and TcdB are monoglucosyltransferases that modify Rho GTPases leading to disorganisation of the actin cytoskeleton, cell-rounding, death of the intoxicated cell and extensive colonic inflammation^4^. The relative contribution of these two major toxins to disease pathogenesis has long been contentious, however, many studies have now clearly demonstrated the importance of TcdB in disease^1, 5–9^ and many strains that produce TcdB but not the other toxins continue to emerge^10, 11^. Targeting TcdB for disease treatment has resulted in the production of a human monoclonal antibody, bezlotoxumab, which reduced rates of recurrent infection in human clinical trials and has recently obtained FDA approval^12^. In support of the approach of targeting TcdB, antibodies against TcdB, but not TcdA, protected piglets from gastrointestinal and systemic signs of CDI when administered intraperitoneally^13^. Moreover, delivery of both anti-TcdA and anti-TcdB neutralising antibodies to either piglets or humans *via* systemic routes was not beneficial compared to anti-TcdB antibodies alone^12, 13^ and administration of anti-TcdA antibodies alone may return adverse clinical outcomes^13^. For these reasons, a universal toxin-based CDI therapeutic must include TcdB as a target and consideration given to the inclusion of a TcdA target.

Rather incongruously, the management of CDI often requires antibiotic administration, usually metronidazole or vancomycin. Although these antibiotics are effective at inhibiting *C. difficile*, they also prevent the re-establishment of the normal, protective microbiota^2^. Consequently, 20–30% of patients experience relapses in *C. difficile* infection after treatment ceases, with many patients suffering multiple relapses^2^. Without doubt, alternative and rationally designed preventive therapies and treatments that do not require the use of antibiotics are required to manage recurrent *C. difficile* disease.

Bovine colostrum is the first milk produced after parturition and is perfectly suited to oral administration; it is therefore ideal for treating gastrointestinal infections^14^. Colostrum provides passive immunity to newborn calves from opportunistic infections and immunisation of dairy cows during gestation with specific antigens results in colostrum containing high concentrations of antigen-specific antibodies. Known as hyperimmune bovine colostrum (HBC), variations of this targeted product, including whole-HBC, immune whey or purified antibodies, have been tested in animals and humans and are effective against many enteric pathogens, including *C*. *difficile* ^15, 16^. Most recently, a whole-HBC product containing antibodies against TcdA and TcdB showed efficacy as a treatment for primary CDI in a gnotobiotic piglet diarrhoea model^17^. *C*. *difficile*-specific HBC products have also previously been shown to be effective at preventing recurrent CDI in human patients^18, 19^. Importantly, colostrum is resistant to gastrointestinal degradation^20^ and, unlike antibiotics, does not adversely disrupt the resident microbiota^17^. This concept is particularly relevant for managing the increasing incidence, severity and recurrence of CDI. As colostrum has the potential to be used as a prevention or treatment of primary disease or recurrence, we examined the use of spore-, vegetative cell- or TcdB-specific colostrum for the prevention and treatment of CDI in a mouse model of infection.

## Results

### *C. difficile*-specific antibodies in colostrum are cross-reactive and able to neutralise TcdB cytotoxicity *in vitro*

**Figure 1:**
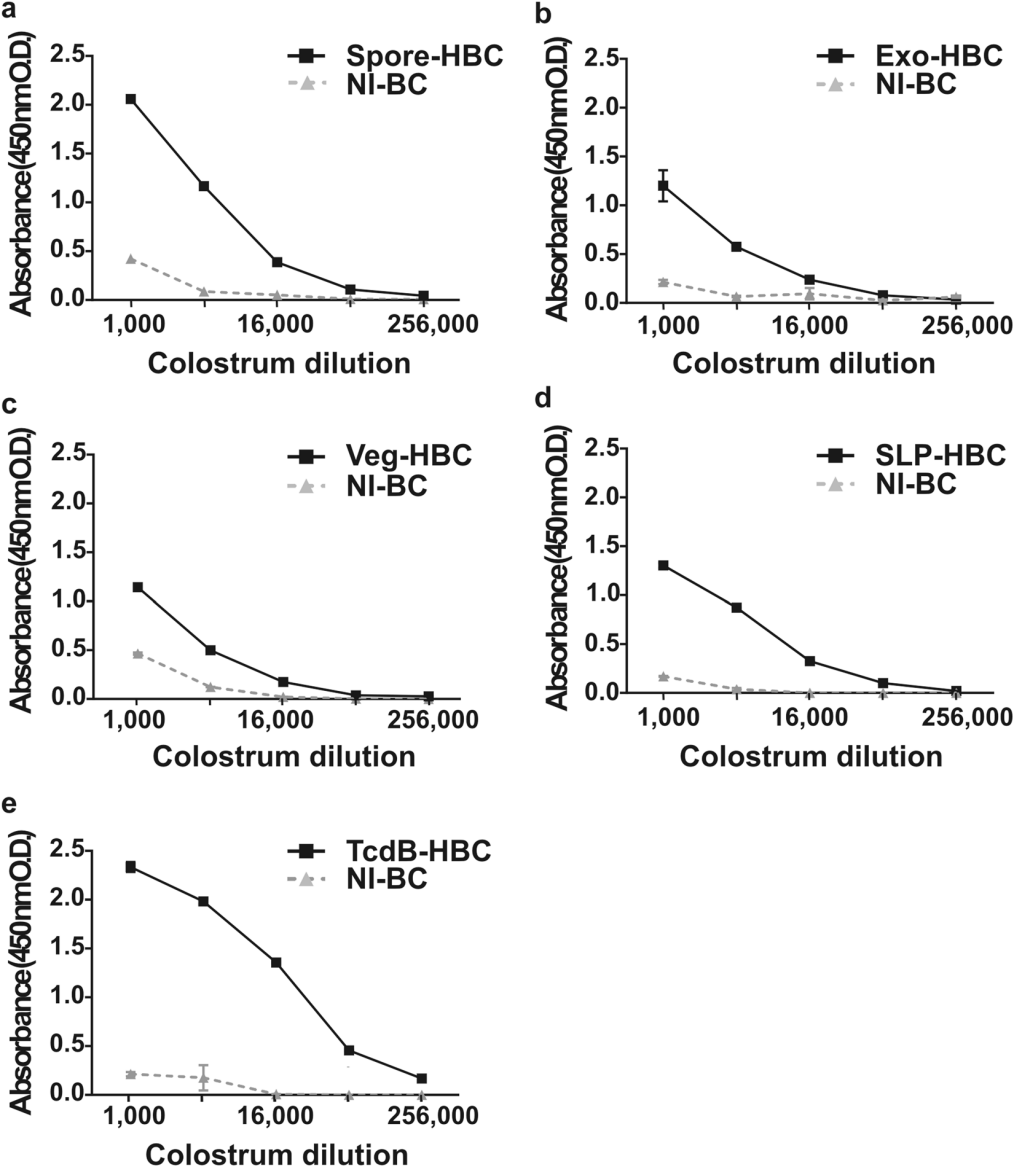
ELISA analysis of *C*. *difficile*-specific antibodies in colostrum from cows immunised with *C. difficile* antigens. ELISA plates were coated with *C*. *difficile* spore, exosporium, vegetative cell, SLP or recombinant TcdB antigens to determine the specific colostrum antibody titres of Spore-HBC (**a**), Exo-HBC (**b**), Veg-HBC (**c**), SLP-HBC (**d**) or TcdB-HBC (**e**), respectively, compared with colostrum from non-immune cows (NI-BC).

**Figure 2:**
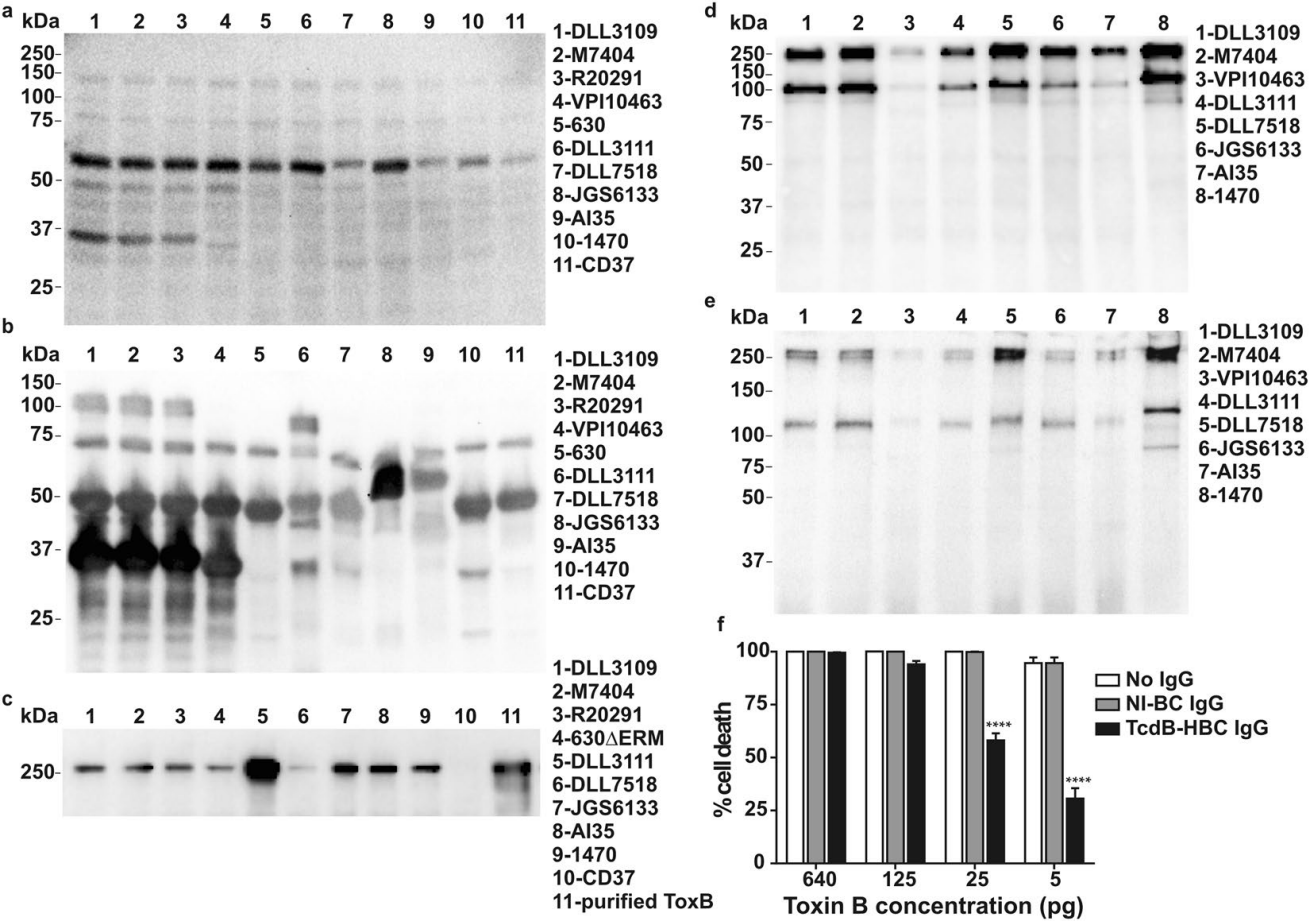
*C. difficile*-specific antibodies in colostrum are cross-reactive and able to neutralise the cytotoxicity of TcdB. *C. difficile* whole cell lysates from a variety of strains were used to detect vegetative cell-specific IgG purified from Veg-HBC (**a**) or SLP-HBC (**b**), respectively. Crude toxin preparations from the panel of strains indicated were used to detect TcdB-specific IgG purified from TcdB-HBC; control lanes contained purified TcdB from an 027 strain (tgcBIOMICS; Lane 11) while a non-toxigenic strain (CD37) was used to show that binding was toxin-specific (Lane 10) (**c**). Exosporium proteins isolated from a variety of clinical and animal isolates of *C. difficile* were used to detect spore-specific IgG purified from Spore-HBC (**d**) or Exo-HBC (**e**), respectively. *C. difficile* strains used are indicated alongside each panel (**a**–**e**). To determine if TcdB-HBC contained IgG capable of neutralising TcdB, purified TcdB from strain VPI10463 (Abcam) was incubated for 60 minutes with either PBS, NI-BC IgG or TcdB-HBC IgG before being added to Vero cell monolayers. After a 24 hour incubation, the percentage of cell death was determined by directly visualising cells for cell rounding (**f**). The experiment was performed in triplicate. Data represent the mean ± SEM and statistical significance was assessed using a two-way ANOVA with a *post* *hoc* Bonferroni multiple comparison test. ****Indicates *P* < 0.0001.

Using platform technologies developed by Immuron Ltd, we produced HBC targeting spores by immunisation of pregnant cows with either inactivated whole spores (Spore-HBC) or an extract of the outermost spore layer, the exosporium (Exo-HBC). We also produced HBC targeting vegetative cells by immunisation with either inactivated vegetative cells (Veg-HBC) or a surface layer protein (SLP) preparation (SLP-HBC). Vegetative cell- or spore-specific vaccines were generated from strain DLL3109, a ribotype 027 isolate. TcdB-specific colostrum (TcdB-HBC) was generated by immunisation with the C-terminal binding domain of TcdB, which was prepared as described elsewhere^21^. *C. difficile*-specific antibody titres in colostrum were determined by ELISA and in all cases were elevated compared to non-immune bovine colostrum (NI-BC) from unvaccinated cows (Fig. 1). Western blotting showed that Veg-HBC and SLP-HBC antibodies cross-reacted with whole cell lysates or SLP from diverse human and animal strains (Fig. 2a,b). TcdB-HBC antibodies cross-reacted with TcdB isolated from culture supernatants of various strains (Fig. 2c). Binding was specific to TcdB as bands were not detected in supernatant from a non-toxigenic strain (Lane 10, Fig. 2c) and were detected in a lane containing TcdB purified from a *C. difficile* 027 strain (tgcBIOMICS; Lane 11, Fig. 2c). Spore-HBC and Exo-HBC antibodies cross-reacted with the exosporium extracted from a panel of strains (Fig. 2d,e). Universal cross-reactivity was seen with each antigen across all strains, suggesting that HBC antibodies may have a broad capacity to combat CDI caused by diverse isolates. Importantly, the TcdB-HBC IgG abolished the cytotoxic activity of purified TcdB, with a significant, dose-dependent, reduction in cell death when 25 pg (*P* < 0.0001) or 5 pg (*P* < 0.0001) of TcdB was incubated with TcdB-HBC IgG compared to TcdB that had been incubated with NI-BC IgG or PBS (Fig. 2f).

### TcdB-specific HBC treats CDI in mice when administered post-infection

**Figure 3:**
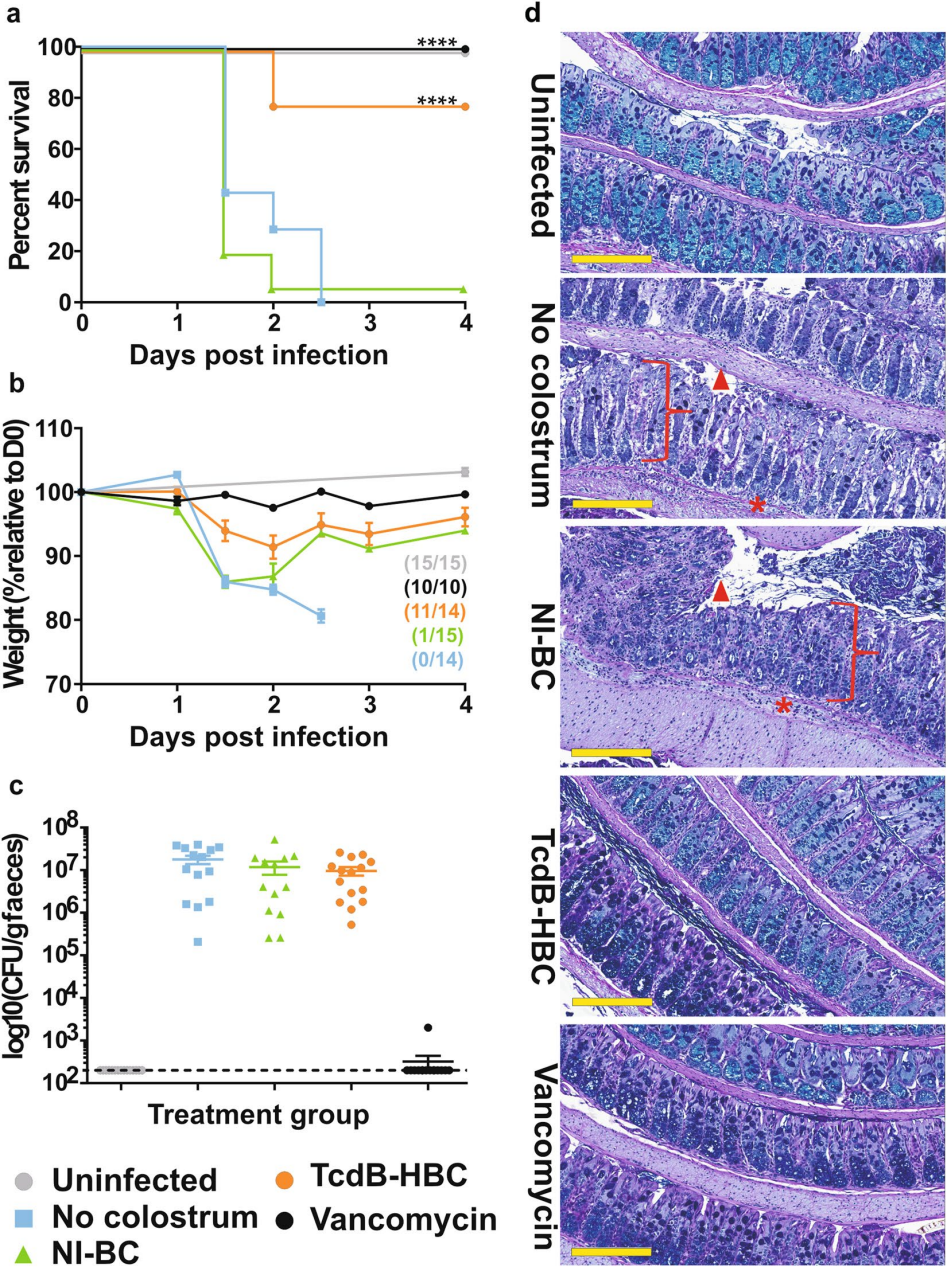
HBC-mediated treatment of *C. difficile*-infected mice. Mice were uninfected or infected with *C. difficile* spores and treated six hours post-infection with NI-BC, TcdB-HBC, vancomycin or were untreated. Mice were monitored daily for survival (**a**) and weight loss (**b**). Weight loss is presented as the % weight relative to the day of infection (day 0 or D0). Numbers in brackets indicate the number of surviving mice at the completion of the experiment compared to (/) the total number of mice at the beginning of the experiment. Faecal spore load was determined 24 hours post-infection and is presented as CFU/gram faeces (log_10_), with each point representing a single mouse (**c**). The limit of detection is represented as a dotted line. Error bars represent the mean ± SEM of n = 10–15 mice. ****Indicates *P* < 0.0001. Representative images of PAS/Alcian blue stained colonic (**d**) tissue from mice. Square brackets ([) indicate crypt hyperplasia, arrow heads (▲) represent epithelial damage and asterisks (*) represent oedema and inflammation. Scale bars represent 200 µm.

The treatment efficacy of the TcdB-HBC was assessed using an infection model. Six hours post-infection, mice were administered NI-BC, TcdB-HBC or vancomycin, the main CDI standard-of-care antibiotic. Untreated and NI-BC-treated mice showed no significant difference in survival, with 0% or 6.7% survival rates (*P* = 0.3178), respectively, while uninfected and vancomycin-treated mice all survived infection (Fig. 3a). By comparison, 78.6% survival was seen in TcdB-HBC-treated mice (Fig. 3a), with TcdB-HBC- (*P* < 0.0001) and vancomycin- (*P* < 0.0001) treated mice showing significantly increased survival rates compared to untreated mice. TcdB-HBC-treated mice showed no statistical difference (*P* = 0.0628) in survival rate compared to vancomycin-treated mice. Uninfected animals did not lose weight (Fig. 3b) and shed no spores, as expected (Fig. 3c), whereas infected but untreated and NI-BC-treated animals lost up to 15-20% of their body weight (Fig. 3b) and shed high spore numbers (~1.5 × 10^7^ CFU/g, Fig. 3c). Infected and vancomycin-treated mice did not lose weight (Fig. 3b) and shed few spores (Fig. 3c), the latter resulting from vancomycin-mediated vegetative cell death and attenuated spore production. By contrast, TcdB-HBC prevented rapid weight loss (Fig. 3b), yet mice shed spores at numbers comparable to both no-treatment and NI-BC groups (Fig. 3c) and developed mild diarrhoea, suggesting that TcdB activity was not completely neutralised or that TcdA was contributing to disease. Mice from all groups that did not survive infection had severe and comparable damage in the colon (Fig. 3d) and caecum (data not shown). Surviving TcdB-HBC-treated mice had less damage in the colon and caecum than NI-BC and no-treatment control mice, however, increased epithelial damage and inflammation was seen compared to uninfected or vancomycin-treated mice (Fig. 3d and data not shown). Collectively, these data provide strong evidence supporting the use of TcdB-HBC to treat CDI.

### Prophylactic administration of TcdB-specific HBC protects mice from CDI

**Figure 4:**
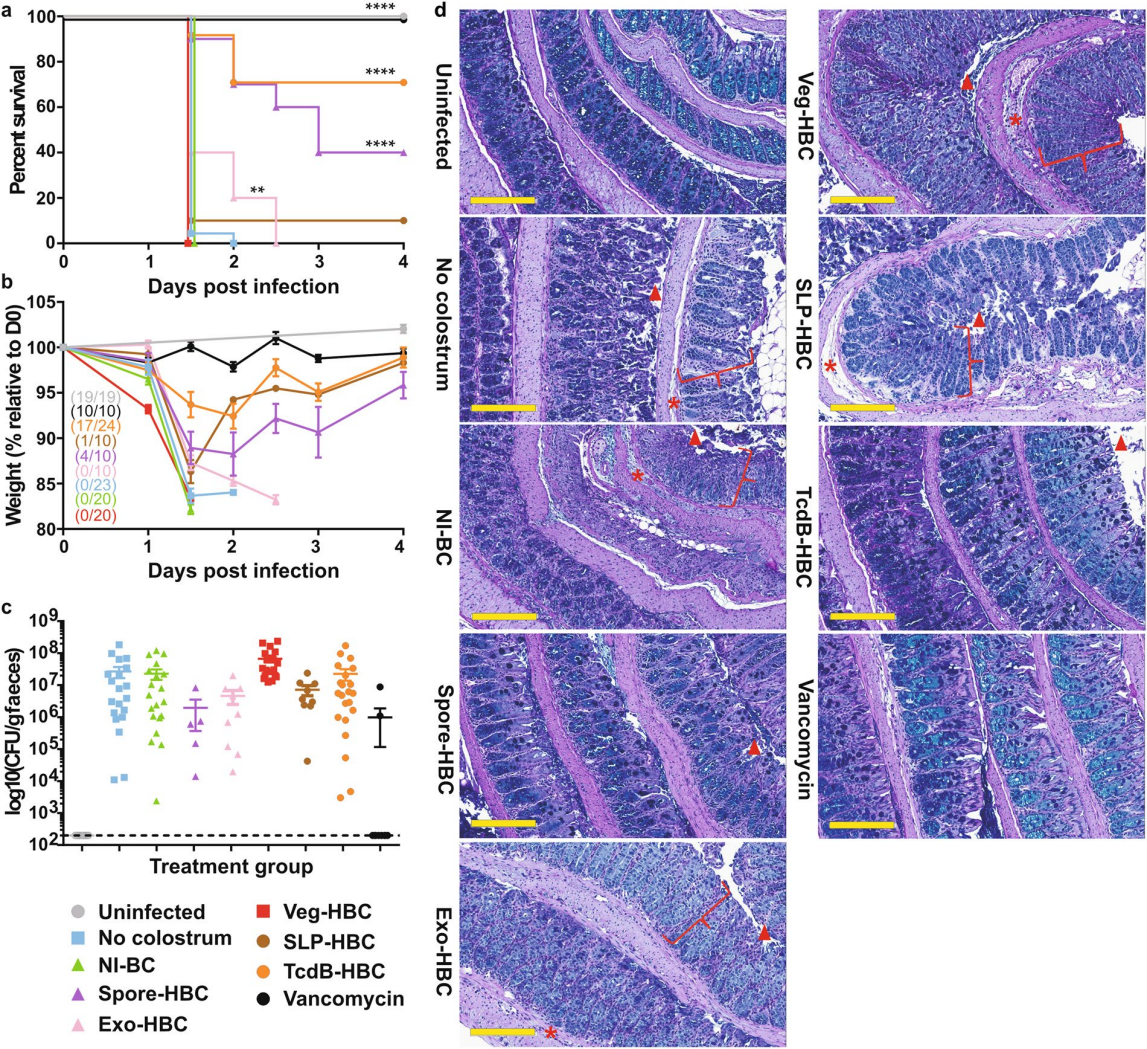
HBC-mediated prevention of *C. difficile* disease in mice. Mice were untreated or were pre-treated for two days with NI-BC, Spore-HBC, Exo-HBC, Veg-HBC, SLP-HBC, TcdB-HBC or vancomycin prior to infection with *C. difficile* spores. Colostrum or vancomycin was administered daily for the duration of the experiment. Uninfected, untreated mice served as controls. Mice were monitored daily for survival (**a**) and weight loss (**b**). Weight loss is presented as the % weight relative to the day of infection (day 0 or D0). Numbers in brackets indicate the number of surviving mice at the completion of the experiment compared to (/) the total number of mice at the beginning of the experiment. Faecal spore load was determined 24 hours post-infection and is presented as CFU/gram faeces (log_10_), with each point representing a single mouse. The limit of detection is represented as a dotted line (**c**). Error bars represent the mean ± SEM of n = 10–24 mice. **Indicates *P* < 0.01, ****Indicates *P* < 0.0001. Representative images of PAS/Alcian blue stained colonic (d) tissue from mice. Square brackets ([) indicate crypt hyperplasia, arrow heads (▲) represent epithelial damage and asterisks (*) represent oedema and inflammation. Scale bars represent 200 µm.

The capacity of TcdB-HBC, Spore-HBC, Exo-HBC, Veg-HBC or SLP-HBC to act prophylactically was also tested. Animals were given access to either water or colostrum *ad libitum* two days prior to infection and throughout the trial. NI-BC-treated and untreated mice succumbed rapidly to infection, with no significant difference in survival observed (*P* = 0.3511; Fig. 4a). Mice that received Spore-HBC lost weight at day 1.5 (Fig. 4b), however, 40% recovered from infection (Fig. 4a) and regained weight (Fig. 4b). Exo-HBC-treated mice did not survive infection, however, death was delayed compared to mice that received no colostrum or NI-BC (Fig. 4a) and weight loss was less rapid (Fig. 4b). Veg-HBC or SLP-HBC-treated mice all succumbed to disease by 1.5–2 days post-infection (Fig. 4a), with rapid weight loss observed during the first 1.5 days (Fig. 4b), similar to untreated mice. Strikingly, 70.8% of TcdB-HBC-treated mice survived infection (Fig. 4a), with less rapid weight loss detected initially and subsequent weight gain reaching levels similar to uninfected and vancomycin-treated groups (Fig. 4b). Spore-HBC- (*P* < 0.0001), Exo-HBC- (*P* = 0.0075), TcdB-HBC- (*P* < 0.0001) and vancomycin- (*P* < 0.0001) treated mice showed significantly increased survival rates compared to untreated mice whereas mice treated with Veg-HBC- (*P* = 0.3511) or SLP-HBC- (*P* = 0.2709) showed no statistical difference in survival compared to untreated mice. When compared to vancomycin-treated mice, Spore-HBC- (*P* = 0.0042), Exo-HBC- (*P* < 0.0001), Veg-HBC- (*P* < 0.0001), or SLP-HBC- (*P* < 0.0001) showed significantly less survival, however, TcdB-HBC-treated mice showed no significant difference in survival (*P* = 0.630). All mice (except vancomycin-treated) shed similar, but variable, numbers of spores 24 hours post-infection (Fig. 4c). Non-surviving mice from all groups had severe damage in the colon (Fig. 4d) and caecum (data not shown). Gut histopathology of surviving TcdB-HBC and Spore-HBC-fed mice looked similar to that of uninfected or vancomycin-treated mice, suggesting that these colostrum preparations prevented severe damage (Fig. 4d). The high survival rate of mice administered TcdB-HBC suggests that targeting TcdB is an effective CDI prophylactic target.

### Prophylactic administration of a mixture of HBC protects mice from CDI

**Figure 5:**
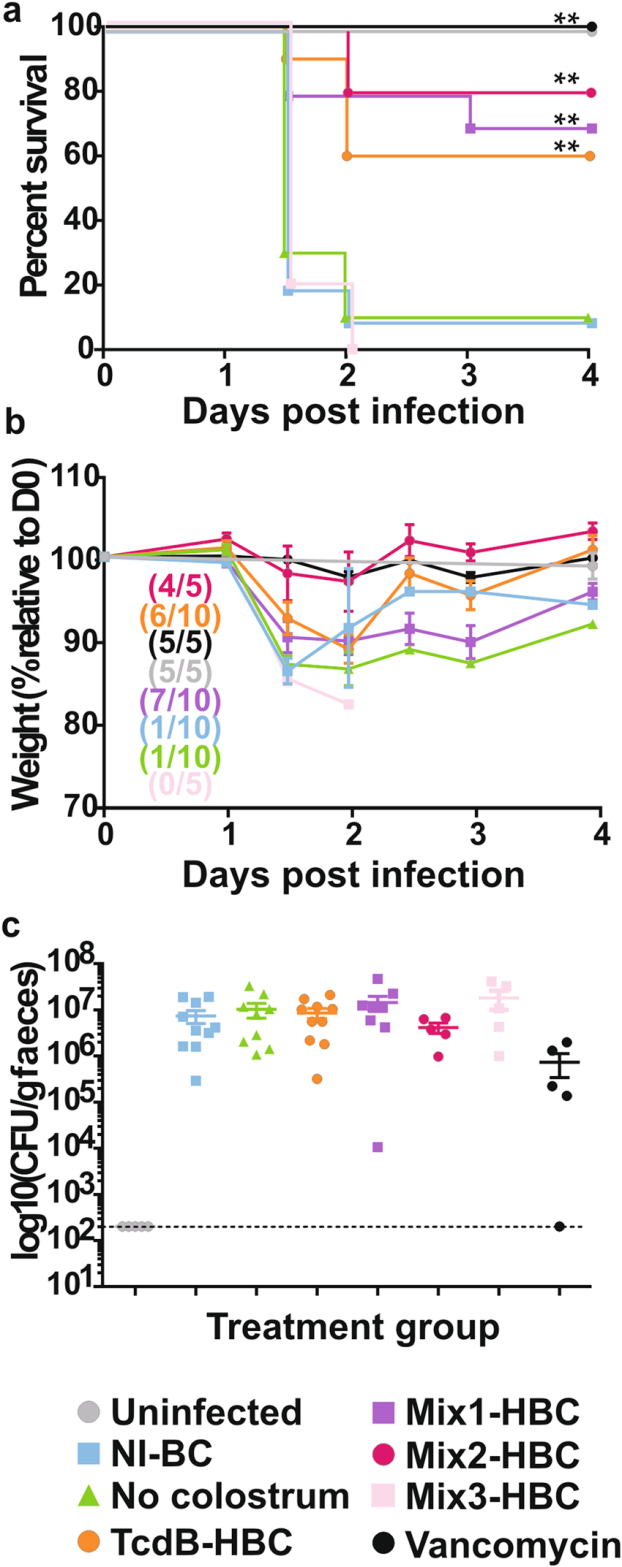
Prevention of primary CDI by HBC mixtures. Mice were untreated (no colostrum) or were pre-treated with NI-BC, TcdB-HBC, Mix1-HBC, Mix2-HBC, Mix3-HBC or vancomycin prior to infection. Uninfected, untreated mice were controls. Mice were monitored daily for survival (**a**) and weight loss (**b**). Weight loss is presented as the % weight relative to the day of infection (day 0 or D0). Numbers in brackets indicate the number of surviving mice at the completion of the experiment compared to (/) the total number of mice at the beginning of the experiment. Faecal spore load is presented as CFU/gram faeces (log_10_), with each point representing a single mouse. The limit of detection is represented as a dotted line (**c**). Error bars represent the mean ± SEM of n = 5–10 mice.

The efficacy of combining colostrum preparations to simultaneously target all stages of the infectious cycle was assessed with either Spore-HBC, Veg-HBC and TcdB-HBC (Mix1-HBC), or Exo-HBC, SLP-HBC and TcdB-HBC (Mix2-HBC) combined in equal ratios (1:1:1). As a control, mice were administered TcdB-specific colostrum that had been diluted 1:3 in non-immune colostrum (Mix3-HBC), which had equivalent amounts of TcdB-specific antibody to Mix2- and Mix3-HBC. Mice were given no treatment or were prophylactically administered either NI-BC, TcdB-HBC, Mix1-HBC, Mix2-HBC or Mix3-HBC. No-treatment and NI-BC-treated mice succumbed to disease by day 2, with no difference in survival (*P* = 0.796; Fig. 5a) and lost up to 15% of their body weight (Fig. 5b), as expected. By comparison, in this experiment, 60% of TcdB-HBC-treated mice survived infection (Fig. 5a), with surviving mice initially losing, then regaining, weight (Fig. 5b). Spore enumeration 24 hours post-infection was similar between all mice (~10^6^ CFU/gram), indicating no colonisation effects (Fig. 5c). Notably, prophylactic administration of Mix1-HBC and Mix2-HBC protected 70% and 80% of mice, respectively, whereas mice receiving Mix3-HBC all succumbed to disease by day 2 (Fig. 5a). Similar to surviving TcdB-HBC-treated mice, Mix1-HBC- and Mix2-HBC-treated mice initially lost but then regained weight (Fig. 5b). Vancomycin- (*P* = 0.0012), TcdB-HBC- (*P* = 0.004), Mix1-HBC- (*P* = 0.0033) and Mix2-HBC- (*P* = 0.0052) treated mice showed significantly increased survival rates compared to untreated mice, whereas TcdB-HBC- (*P* = 0.1174), Mix1-HBC- (*P* = 0.6998) and Mix2-HBC- (*P* = 0.4292) treated mice showed no statistical difference in survival compared to vancomycin-treated mice, suggesting that colostrum treatment is comparable to the standard-of-care antibiotic for CDI. Collectively, this data suggests that there is added benefit in combining TcdB-specific colostrum with colostrum that targets spores and vegetative cells. Although not significant, the mixed-colostrum products yielded a higher level of protection in mice compared to TcdB-HBC alone and thus subsequent experiments utilised a colostrum mixture.

### A mixture of HBC protects mice from disease recurrence

**Figure 6:**
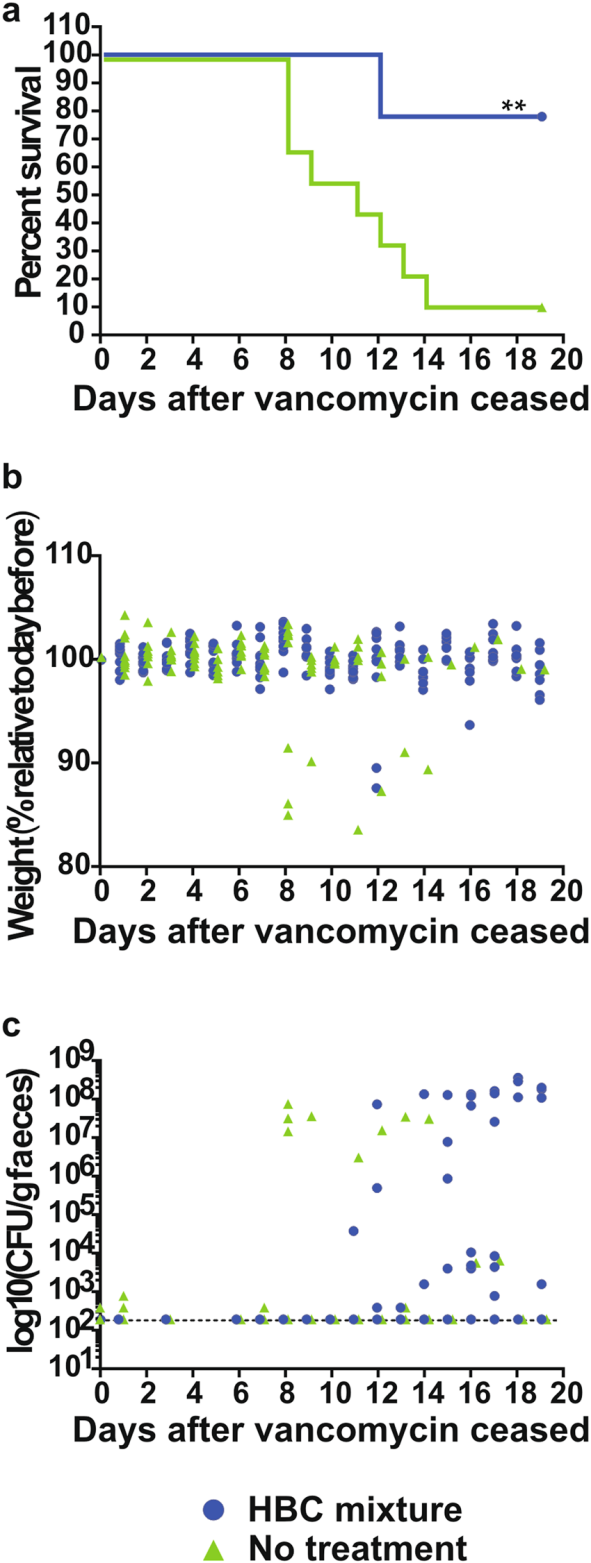
Prevention of recurrent CDI by HBC mixtures. Infected mice were orally treated with vancomycin prior to receiving either vancomycin or vancomycin and a mixture of Exo-HBC, SLP-HBC and TcdB-HBC. After vancomycin treatment ceased, mice either received no treatment or the HBC mixture. Mice were monitored daily for survival (**a**) and weight loss (**b**). Weight loss is presented as the % weight relative to the day before, with each point representing a single mouse. Faecal spore load was determined daily after cessation of vancomycin treatment and is presented as CFU/gram faeces (log_10_) as described above (**c**). Error bars represent the mean ± SEM of n = 9 mice. **Indicates *P* < 0.01.

Disease recurrence occurs in 20-30% of patients and many current strategies are targeting this important disease aspect^1^. We therefore tested a combined Exo-HBC, SLP-HBC and TcdB-HBC product in a disease relapse model. Most mice receiving no-colostrum treatment succumbed to disease 8–14 days after vancomycin cessation, with a survival rate of 11.1% (Fig. 6a). Severe diarrhoea and weight loss was seen (Fig. 6b) and high spore numbers were detected on the day animals succumbed (Fig. 6c). By contrast, colostrum-treated mice had a significantly higher survival rate of 77.8% (*P* = 0.0027; Fig. 6a) and lost little weight (Fig. 6b). Although the surviving mice from this group began shedding spores (Fig. 6c) and had mild diarrhoea, they did not succumb to disease, suggesting that colostrum antibodies did not prevent bacterial growth and colonisation, but effectively neutralised TcdB activity. Overall, this work suggests that an Exo-HBC, SLP-HBC and TcdB-HBC combination product effectively prevents *C*. *difficile* disease relapse.

## Discussion

We have developed the first oral whole-HBC immunotherapeutic product specifically engineered to neutralise the activity of only one of the *C*. *difficile* toxins, TcdB. Until now most vaccines and immunotherapeutics have focussed on TcdA alone or have combined TcdA and TcdB, however, many pathogenic strains do not produce TcdA whereas almost all produce TcdB^11^. The importance of TcdB, particularly in severe intestinal damage^22^ and severe^8^ and systemic disease^13, 22^, has been established, For these reasons, TcdB was selected as a key target for the development of *C. difficile* colostrum-based therapeutics, together with vegetative cell and spore targets that represent all important infectious cycle components.

The use of *C. difficile*-specific colostrum as a prevention or treatment for CDI has yielded promising results when tested in various animal models and in human clinical trials. Colostrum directed against *C. difficile* culture supernatant or purified TcdA was shown to prevent the enterotoxic effect of toxins in a rat ileal loop model^23^. Most recently, whole colostrum that was generated by vaccinating cows with both recombinant TcdA and TcdB was shown to successfully treat CDI in a gnotobiotic piglet model when administered after the onset of mild diarrhoea^17^. Prophylactic administration of colostrum immunoglobulin concentrate containing antibodies against *C. difficile* culture filtrate (with both TcdA and TcdB present) protected hamsters from CDI^24^. Moreover, treatment of hamsters with a bovine milk product (MucoMilk) that contained high titres of sIgA, raised against whole *C. difficile* cells and toxoid prepared from bacterial culture filtrate, prevented disease^18^. This same product was shown to prevent disease relapse in human patients^18, 19^, while bovine colostrum containing antibodies raised against *C. difficile* vegetative cells was shown to be as effective as metronidazole in treating recurrent CDI in humans^25^. It has also been shown that *C. difficile*-specific colostrum antibodies survive transit through the human gastrointestinal tract and retain their ability to neutralise toxin activity^20, 26^. As colostrum has the potential to be used for the prevention or treatment of primary disease or recurrence, we examined the use of spore-, vegetative cell- or TcdB-specific colostrum for the prevention and treatment of CDI in a mouse model of infection.

The TcdB-HBC product developed here showed excellent efficacy, supporting our hypothesis that targeting TcdB is beneficial in preventing and treating primary CDI. Dilution of TcdB-specific colostrum with non-immune colostrum at a ratio of 1:3 reduced the efficacy of the TcdB colostrum, but a similar dilution, using a combination of spore and vegetative cell colostrum, in which the ratio of TcdB antibody remained 1:3, resulted in enhanced survival in mice, suggesting that these antibodies acted synergistically to provide superior protection against CDI. Importantly, our product combining TcdB-, SLP- and Exo-HBC reduced disease relapse in mice by 66.7%. Supporting our approach of targeting TcdB, intravenous infusion of the monoclonal TcdB antibody product bezlotoxumab (Merck) during a phase III clinical trial reduced disease relapse from 28% to 17% in MODIFY I and 26% to 16% in MODIFY II compared to placebo^12^. The use of bovine milk or modified colostrum to reduce CDI relapse rates in human clinical trials^18, 19, 25^ also supports the efficacy of a colostrum product. However, the antibodies used in these studies were generated against vegetative cells alone or in combination with TcdA- and TcdB-containing culture supernatants, and whole HBC was not investigated.

Although the development of colostrum based immunotherapeutics for CDI treatment is not a new concept, our study is the first to clearly show a reduction in recurrence with a defined colostrum product. We are the first to test a colostrum-based therapy in a mouse model of CDI as well as being the first to show that colostrum antibodies targeting TcdB alone are sufficient for preventing and treating disease. We have also shown for the first time that combining different batches of colostrum, containing antibodies against individual *C. difficile* components, generates a product that is superior to targeting a single antigen alone. Finally, our results have proven the efficacy of this type of therapy in three different aspects of disease: prevention and treatment of primary disease and prevention of recurrent CDI, the latter of which represents an important disease aspect currently being targeted in the development of many diverse therapeutic products. For our product to progress from pre-clinical studies to the clinic, the delivery and dose of colostrum required to prevent disease in humans needs to be determined. Although colostrum inherently protects antibodies from gastrointestinal tract degradation, allowing transit to the colon^26^, encapsulation or sustained-released formulations may improve product efficacy by increasing the amount and longevity of the antibodies in the colon.

The period of recovery of the microbiota following antibiotic treatment for CDI is hypothesised to correspond to the window of susceptibility to recurrence^27^. The systemic administration of monoclonal TcdA and TcdB antibodies to mice facilitates the normalisation of the gut microbiota during CDI while reducing toxin-mediated damage^28^, and may prevent recurrent CDI by reducing clinical disease manifestations until the microbiota is replenished and colonisation resistance to *C. difficile* is restored^27^. We hypothesise that HBC could be orally administered to patients in parallel with standard-of-care antibiotics, and continued after the cessation of antibiotic treatment to prevent recurrent infection. Colostrum allows transit of the antibodies to the colon^26^, where they can neutralise toxin activity directly at the site of infection, allowing a healthy microbiota to be restored and repair of the damaged gut to proceed. By comparison, it is unknown how systemically-delivered monoclonal antibodies protect against toxins located in the large intestine, although it has been suggested that toxin-mediated damage to the gut mucosa allows these antibodies to leak into the gut lumen and then neutralise the toxins, protecting the mucosa from further toxin-induced damage^29^. Extensive toxin-mediated mucosal damage leading to a leaky gut permits extra-intestinal dissemination of toxins and other lumen components, resulting in systemic disease in animal models^30^. Orally delivered toxin-neutralising antibodies could reduce gut leakage and subsequent systemic effects by lessening the severity of gut damage and decreasing the extra-intestinal dissemination of toxins or other damaging factors.

Our work provides strong proof-of-principle evidence that HBC for the prevention and treatment of *C*. *difficile* infections is effective. Oral delivery of antibodies allows easy administration to patients as a treatment for primary or relapsing disease, while the low cost of production (USD$1/gram of colostrum polyclonal antibodies (Immuron Ltd, personnel communication, December, 2016) compared to USD$100/gram for the equivalent production of monoclonal antibodies^31^) may permit their use prophylactically. Colostrum IgG antibodies can be readily generated in large amounts, with an average of 200 grams of pure IgG extracted from 5 kilograms of colostrum produced from each dairy cow (Immuron Ltd, personnel communication, April, 2017). These features make HBC an ideal product for the treatment of *C*. *difficile* and other enteric pathogens, particularly those that are antibiotic resistant, and the successful use of HBC for *Escherichia coli*, *Cryptosporidium*, rotavirus and *Shigella flexneri* infections^16^ supports this approach.

## Methods

### Bacterial culture conditions and strains

*C. difficile* strains were cultured on HIS agar [heart infusion (HI) (Oxoid) containing 1.5% glucose, 0.1% (w/v) L-cysteine, 1.5% (w/v) agar] in an anaerobic chamber (Don Whitley Scientific) at 37 °C. To prepare vegetative cells, spores were germinated on HIS-T agar [HIS agar containing 0.1% (w/v) sodium taurocholate (New Zealand Pharmaceuticals)], subcultured and inoculated into HIS-T broth. A mid-exponential culture (OD_600_ 0.40–0.70) was used to inoculate another HIS-T broth. A 1:100 dilution of this culture (OD_600_ of 0.50) was used to inoculate a final HIS-T broth and grown to an OD_600_ of 0.50–0.70. The cells were washed 3 times with PBS (3200 × *g* for 20 minutes at 4 °C) and sonicated to prepare whole cell lysates for use in Western blotting. For immunisations, cells were fixed using 10% (v/v) formalin in PBS overnight (4 °C), then washed 3 times with PBS. To prepare spores, strains were subcultured on HIS-T agar and inoculated into 500 mL Tryptone Yeast (TY) broth [3.0% tryptone, 2.0% yeast extract and 0.1% sodium thioglycolate] and grown anaerobically (37 °C) for 10 days. Spores were harvested by centrifugation at 10,000 × *g* for 20 minutes at 4 °C and washed five times with chilled dH_2_O. To inactivate spores for vaccinations, spores were exposed to gamma irradiation for 200 hours (12, 840 Gray) followed by overnight treatment with 10% (v/v) formalin in PBS at 4 °C and three washes with PBS. For mouse infections, spores were resuspended in PBS containing 0.05% tween-80 and heat shocked at 65 °C for 20 min. Strains used in this study were: DLL3109 (TcdA^+^TcdB^+^)^32^, M7404 (TcdA^+^TcdB^+^)^33^, R20291 (TcdA^+^TcdB^+^)^34^, VPI10463 (TcdA^+^TcdB^+^)^35^, 630∆ERM (TcdA^+^TcdB^+^)^36^, DLL3111 (TcdA^+^TcdB^+^), DLL7518 (TcdA^−^TcdB^+^), JGS6133 (TcdA^+^TcdB^+^)^37^, AI35 (TcdA^−^TcdB^+^)^38^, 1470 (ATCC 43598; TcdA^−^TcdB^+^)^39^, and CD37 (TcdA^−^TcdB^−^)^40^.

### Preparation of exosporium

Exosporium from the spore surface was prepared as previously described^41^. Briefly, spores were resuspended in extraction buffer (50 mM Tris-HCl, pH 10; 8 M Urea; 2% (v/v) 2-mercaptoethanol) and heated (15 minutes, 90 °C) prior to centrifugation at 13, 000 × *g* for 10 minutes at room temperature (RT). The supernatant was dialysed against PBS overnight using 6000–8000 MWCO dialysis tubing. For immunisations, the exosporium preparation was fixed using 10% formalin (v/v) in PBS (8 hours, 4 °C) prior to further dialysis and concentrated to 1 mg/mL in PBS using 10,000 MWCO centrifugal filters.

### Preparation of surface layer proteins (SLP)

SLP from the vegetative cell surface was prepared as described previously^42^. Briefly, overnight cultures of vegetative cells were harvested by centrifugation at 3200 × *g* for 20 minutes (4 °C). Cells were washed once with PBS, then resuspended in 0.04 volumes of 0.2 M glycine pH 2.2 and incubated, mixing end over end, for 30 minutes at RT. The sample was centrifuged at 16, 200 × *g* for 10 minutes and the supernatant containing S-layer proteins was collected. The supernatant was neutralised using 2 M Tris pH 9.0 and dialysed overnight in PBS. For immunisations, the SLP preparation was fixed, dialysed and concentrated as before.

### Partial purification of *C. difficile* toxins from culture media

A three day old 500 mL TY broth culture was centrifuged (10,000 × *g*, 15 min, 4 °C) and the toxin-rich supernatant was filter sterilised (0.2 μm filter) and concentrated ten-fold using a 100,000 MWCO concentrating cassette. The supernatants were dialysed using PBS, filter sterilised (0.2 μm filter) and adjusted to 1 mg/mL in PBS.

### Construction of a C-terminal TcdB expression plasmid

Using *C. difficile* strain 630 genomic DNA as a template, a PCR product corresponding to the C-terminal end of TcdB was amplified as previously described^21^ and cloned into pGEM-T Easy (Promega); the resulting vector was used as the template for further PCR. The TcdB fragment was amplified from this vector using DLP865 (5′ ATGCCATATGGAAGAAAATAAGGTGTCACAAG 3′) and DLP866 (5′ ATGCCTCGAGTTGAGCTGTATCAGGATCA 3′), which are the same as OL169 and OL170^21^, except that the BamHI and EcoRI restriction sites were changed to NdeI and XhoI to facilitate cloning into pET30b. This final vector, pDLL217, was introduced into *E. coli* BL21 (DE3) cells for protein expression.

### Recombinant protein expression and purification

*E. coli* cells were cultured overnight in Terrific broth supplemented with 50 µg/mL of kanamycin at 37 °C with shaking on a rotating platform at 200 rpm. Bacteria from these cultures were inoculated into 200 mL of Terrific broth (1:20 dilution), which was grown until the OD_600nm_ reached 1.2. Expression was induced by adding 0.1 mM isopropyl β-D-1-thiogalactopyranoside (IPTG; Promega) for five hours (37 °C), with shaking at 250 rpm. Cells were pelleted by centrifugation at 2,500 × *g* for 15 minutes at 4 °C, resuspended in lysis buffer [100 mM sodium phosphate pH 7.4, 0.15 M NaCl] and frozen at −80 °C. For protein purification, cells were washed twice in wash buffer I [100 mM sodium phosphate pH 7.4, 0.15 M NaCl, 1% (v/v) Triton X-100, 1 mM 2-mercaptoethanol] followed by one wash with wash buffer II [100 mM sodium phosphate pH 7.4, 0.15 M NaCl, 1 mM 2-mercaptoethanol]. Cell pellets were resuspended in resuspension buffer [100 mM sodium phosphate pH 7.4, 0.15 M NaCl, 8 M urea, 10 mM 2-mercaptoethanol] and incubated at 25 °C for 1 hour with shaking at 100 rpm. Cell pellets were compacted by centrifugation at 30, 000 × *g* for 20 minutes at 10 °C and the filtered insoluble fraction was loaded onto a His Trap column (GE Healthcare Biosciences) at 1 mL/minute. The column was washed with His Trap buffer [100 mM sodium phosphate pH 7.4, 0.15 M NaCl, 20 mM imidazole, 8 M urea, 1 mM 2-mercaptoethanol] and the protein eluted from the column in 5 column volumes using His Trap elution buffer [100 mM sodium phosphate pH 7.4, 0.15 M NaCl, 0.5 M imidazole, 8 M urea, 1 mM 2-mercaptoethanol]. Eluted protein was loaded onto a Hi Prep 26/10 desalting column (GE Healthcare Biosciences) and 2 mL fractions collected in desalting buffer [100 mM sodium phosphate pH 7.4, 0.15 M NaCl, 8 M urea]. Fractions containing the protein of interest were pooled, diluted to 30 mL with desalting buffer and dialysed (4 °C) against a series of buffers (50 mM Tris-HCl pH 8.0, 100 mM NaCl, 10% (v/v) glycerol) containing successively decreasing amounts of urea (4 M, 2 M, 1 M, 0 M) using 10 kDa cut-off dialysis tubing. The protein was concentrated to 1 mg/mL and frozen at −80 °C.

### Immunisations

Animal handling and experimentation was performed in accordance with Victorian Government guidelines (Department of Economic Development, Jobs, Transport & Resources. All experimental protocols were approved by the Immuron Ltd animal ethics committee (ethics numbers A17 and A18). Pregnant Holstein dairy cows received three intramuscular vaccinations beginning eight weeks prior to the expected calving date. Immunisations were administered every two weeks, with the final dose administered four weeks prior to calving. Vaccines were prepared by emulsifying 1 mL of each antigen with the same volume of Montanide ISA206VG adjuvant (Tall Bennett). Cows received 1 × 10^9^ spores per dose (3 cows), 2 × 10^10^ vegetative cells per dose (3 cows), 0.5 mg (6 cows) or 1 mg of TcdB (3 cows) per dose, 0.5 mg of exosporium per dose (3 cows), or 0.5 mg of SLP per dose (3 cows).

### Colostrum preparation and purification of IgG antibody

Colostrum was collected up to 12 hours post calving. Fat was removed by centrifugation at 10,000 × *g* for 30 minutes (4 °C) and the colostrum pasteurised at 63.5 °C for 30 minutes. Following rapid chilling on ice, the colostrum was centrifuged at 10,000 × *g* for 30 minutes at 4 °C and concentrated to approximately 60% of the original volume using a 30, 000 MWCO cassette. The original volume of colostrum was restored by the addition of dH_2_O and diafiltration performed by concentrating the colostrum again to approximately 60% of the original volume. Concentrated colostrum was then freeze dried at −80 °C and reconstituted to 10% (w/v) in dH_2_O. IgG antibody was purified from the colostrum using a protein G column (GE Healthcare) according to the manufacturer’s instructions.

### Measurement of *C. difficile*-specific IgG levels in colostrum

*C. difficile*-specific IgG binding titres in whole HBC were measured by enzyme-linked immunosorbent assay (ELISA). Wells of a 96-well Maxisorp plate were coated with 100 µl of the immunisation antigens [recombinant TcdB (1 μg/mL), vegetative cells (10^6^/mL), SLP (1 µg/mL), spores (10^5^/mL) or exosporium (1 µg/mL)] in 50 mM carbonate-bicarbonate buffer [pH 9.6] and incubated overnight (4 °C). Plates were washed five times with PBS-0.1% (v/v) tween-20 (PBST), blocked for two hours (RT) with 5% (w/v) casein in PBS and washed again. Reconstituted colostrum was diluted 1:250 in 0.5% (w/v) casein in PBST. Five four-fold serial dilutions were performed and 100 μl of colostrum was added to triplicate wells and incubated at RT for two hours. As a background control, wells received 0.5% casein in PBST. Plates were washed and 100 μl of goat anti-bovine IgG-peroxidase conjugate, diluted 1:2000 in PBST, was added to each well and incubated for one hour (RT). Following washing, colour was developed by the addition of 100 μl/well of 3,3′,5,5;-tetramethylbenzidine (TMB). The reaction was stopped by 50 μl/well of 2 M H_2_SO_4_. Absorbance was read at 450 nm. The endpoint titre was determined as the reciprocal of the highest analyte dilution that gave a value two times higher than the mean background control reading.

### SDS-PAGE and Western blotting

Proteins (10 µg) were separated by 12.5% (v/v) sodium dodecyl sulfate-polyacrylamide gel electrophoresis (SDS-PAGE) and transferred onto a nitrocellulose membrane. Proteins were detected using purified IgG colostrum antibodies (1:500 dilution of 30 mg/mL stock), followed by a goat anti-bovine IgG-HRP antibody (1:2000 dilution). Antigen-antibody complexes were detected using a Western Lightning Chemiluminescence reagent kit and visualised using a Chemidoc system (Biorad).

### TcdB neutralisation assays

Vero cells were cultured as described^8^. For neutralisation assays, cells were seeded in 96-well plates at 1 × 10^4^ cells/well and incubated for 24 hours at 37 °C in 5% CO_2_. Purified TcdB from strain VPI10463 (Abcam) was diluted in MEM-α containing 1% (v/v) FCS to 6.4 ng/mL, 1.25 ng/mL, 0.25 ng/mL and 0.05 ng/mL. Toxin preparations were incubated (60 minutes) with either PBS or a 1:20 dilution of a 20 mg/mL stock of purified non-immune IgG or TcdB-specific IgG. The toxin or toxin-antibody complexes were then added to the Vero cells (100 μl/well), resulting in final toxin concentrations of 640 pg, 125 pg, 25 pg and 5 pg. Cells were incubated (24 hours) before being observed by microscopy (Olympus 1X71 inverted microscope) and scored for cytopathic effect (CPE). Percentage cell death was determined as the percentage of cells undergoing cell rounding.

### Murine models for disease prevention and treatment

Animal handling and experimentation was performed in accordance with Victorian State Government regulations and approved by the Monash University Animal Ethics Committee (Monash University AEC no. SOBSB/M/2010/25 and MARP/2014/134). Male, 6–7 week old, C57BL/6 J mice (Walter and Eliza Hall Institute of Medical Research, Melbourne, Australia) were pre-treated with an antibiotic cocktail in the drinking water for seven days as described^22^, followed by two days of cefaclor alone. Antibiotic treatment ceased one day prior to infection with *C. difficile* strain DLL3109 (10^3^ spores/mouse) by oral gavage. For colostrum prophylaxis studies, mice were administered colostrum in the drinking water two days prior to infection, continuing for the trial duration. For treatment studies, mice received 200 µl of colostrum (10% w/v) or 100 µl of vancomycin (6 mg/mL) by oral gavage six hours post-infection followed by immediate access to colostrum (10% w/v) or vancomycin (0.4 mg/mL) in the drinking water. Mice were monitored twice daily for disease signs (weight loss, behavioural and physical changes, and diarrhoea). Faecal pellets were collected 24 hours post-infection and resuspended in PBS (100 mg/mL), heat shocked (30 minutes, 65 °C) and plated for spore enumeration as described^43^. Animals were humanely killed by CO_2_ overdose or cervical dislocation when defined endpoints were met as previously defined^22^. The colon and caecum from each mouse were swiss-rolled^44^ and fixed in 4% (w/v) phosphate buffered formaldehyde solution, sectioned transversely and stained with Periodic Acid Schiffs (PAS) and Alcian Blue. Tissues were scanned using Aperio ScanScope and imaged using Aperio ImageScope and assessed for histopathological damage as described^22^.

### Murine model for prevention of disease recurrence

Mice were pre-treated with antibiotics and infected as above. Ten hours following infection, all mice were administered 100 µl of vancomycin (6 mg/mL) by oral gavage and then given access *ad libitum* to either vancomycin (0.4 mg/mL) alone in the drinking water (group A) or colostrum (15% (w/v)) containing 0.4 mg/mL of vancomycin (group B). These solutions were replenished daily. Mice were monitored daily for signs of infection and faecal samples were enumerated for the presence of spores. Once levels of *C. difficile* in the faeces reached undetectable levels, vancomycin treatment ceased (day 8) and mice were housed individually to assess disease relapse. At this point, mice were given either plain water (group A) or colostrum (group B). Mice were then weighed and faecal samples collected daily to detect spore shedding and disease relapse. Mice were humanely euthanised according to animal ethics guidelines if they lost 10% body weight in 24 hours or met other disease criteria as previously defined^22^.

### Statistical Analysis

Statistical analysis was performed using Prism 6 software (GraphPad Software). The *in vitro* toxin neutralisation assay was analysed by two-way ANOVA with a *post* *hoc* Bonferroni multiple comparison test. The Kaplan-Meier survival curves were assessed using a log-rank (Mantel-Cox) test. Differences in data values were considered significant at a *P* value of <0.05.

### Data Availability

All data generated or analysed during this study are included in this published article.
